# Tongguan Capsule Mitigates Post-myocardial Infarction Remodeling by Promoting Autophagy and Inhibiting Apoptosis: Role of Sirt1

**DOI:** 10.3389/fphys.2018.00589

**Published:** 2018-05-22

**Authors:** Shuai Mao, Peipei Chen, Ting Li, Liheng Guo, Minzhou Zhang

**Affiliations:** ^1^Key Discipline of Integrated Chinese and Western Medicine, Second Clinical College, Guangzhou University of Chinese Medicine, Guangzhou, China; ^2^Department of Critical Care Medicine, Guangdong Provincial Hospital of Chinese Medicine, Guangzhou, China; ^3^Cardiovascular Institute, Stanford University School of Medicine, Stanford, CA, United States

**Keywords:** cardiac remodeling, Tongguan capsules, autophagy, apoptosis, Sirt1

## Abstract

Left ventricular (LV) adverse remodeling and the concomitant functional deterioration contributes to the poor prognosis of patients with myocardial infarction (MI). Thus, a more effective treatment strategy is needed. Tongguan capsule (TGC), a patented Chinese medicine, has been shown to be cardioprotective in both humans and animals following ischemic injury, although its precise mechanism remains unclear. To investigate whether TGC can improve cardiac remodeling in the post-infarct heart, adult C57/BL6 mice underwent coronary artery ligation and were administered TGC or vehicle (saline) for 6 weeks. The results demonstrated that the TGC group showed significant improvement in survival ratio and cardiac function and structure as compared to the vehicle group. Histological and western blot analyses revealed decreased cellular inflammation and apoptosis in cardiomyocytes of the TGC group. Furthermore, TGC upregulated the Atg5 expression and LC3II-to-LC3I ratio but downregulated autophagy adaptor p62 expression, suggesting that TGC led to increased autophagic flux. Interestingly, with the administration of 3-methyladenine, an autophagy inhibitor, in conjunction with TGC, the aforesaid effects significantly decreased. Further mechanistic studies revealed that TGC increased silent information regulator 1 (Sirt1) expression to reduce the phosphorylation of the mammalian target of rapamycin and its downstream effectors P70S6K and 4EBP1. Moreover, the induction of Sirt1 by TGC was inhibited by the specific inhibitor EX527. In the presence of EX527, TGC-induced autophagy-specific proteins were downregulated, while apoptotic and inflammatory factors were upregulated. In summary, our results demonstrate that TGC improved cardiac remodeling in a murine model of MI by preventing cardiomyocyte inflammation and apoptosis but enhancing autophagy through Sirt1 activation.

## Introduction

Recent advances in the treatment of myocardial infarction (MI), such as early coronary reperfusion therapy and established standards medical care, have contributed to increased preservation of a viable myocardium and notably reduced the mortality rates (Cuenin et al., 2017). However, the parallel increase of the prevalence of and mortality from pathological myocardial remodeling and heart failure following MI has emerged as a growing challenging public health problem (Sharp et al., 2017). Currently, therapeutic strategies to prevent adverse left ventricular (LV) remodeling and heart failure after MI remain limited (Hassell et al., 2017). Thus, the attenuation of cardiac remodeling has long been the focus for efficient strategies to improve patients’ post-infarct prognosis.

Studies on cardiomyocytes apoptosis and autophagy in the pathophysiological process of post-infarct adverse cardiac remodeling may provide novel treatment or prevention strategies (Kunapuli et al., 2006). Following MI, apoptosis occurs early in the remote myocardium and has been generally considered a potent inducer contributing to myocardial remodeling (Bharti et al., 2015). However, recent evidence has depicted an indefinite role of autophagy which plays an important part in maintaining intracellular hemostasis and function by degrading and recycling non-functional protein aggregates and scavenging damaged organelles at the basal levels (Levine and Klionsky, 2004). In addition, autophagy can be further induced in the heart by stress, such as acute and chronic ischemia, heart failure, and aging (Thapalia et al., 2014; Riquelme et al., 2016; Shirakabe et al., 2016). Cardiac specific loss of autophagy through a genetic deficiency of autophagy-related 5 (Atg5) was shown to cause cardiomyopathy, LV dilatation, and contractile dysfunction (Nakai et al., 2007). This is in line with another chronic cardiac ischemic pig model, which showed that repetitive myocardial stunning induced autophagy with less apoptosis followed by fully recovered heart function. These data suggest that autophagy may be essential for survival of the hibernating myocardium (Yan et al., 2005). On the contrary, inhibition of autophagy with 3-methyladenine (3MA) reduced autophagosome formation and subsequent autophagic cell death of rat cardiomyocyte-derived H9c2 cells during glucose deprivation (Aki et al., 2003). However, whether it is necessary for the survival of or detrimental to cardiomyocytes, the precise role of autophagy in pathological remodeling remains to be elucidated.

**FIGURE 1:**
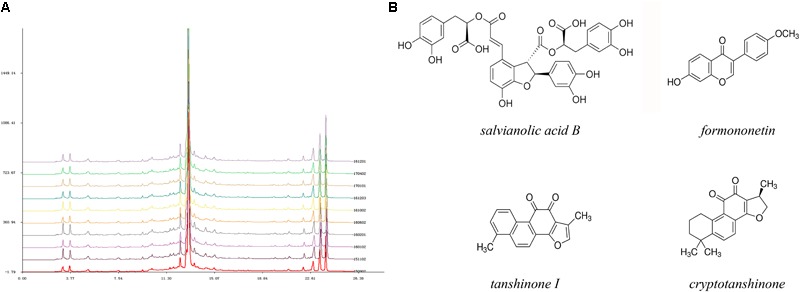
High-performance liquid chromatography fingerprint analysis of Tongguan capsules. **(A)** HPLC analysis identified nearly 15 active chemical components, including salvianolic acid B, fermlononetin, tanshinone I, cryptotanshinone and tanshinone IIA. The similarity of 10 batches of TGC and the reference fingerprint was greater than 99.72%. **(B)** Structure of representative components.

From the perspective of traditional Chinese medicine (TCM), the deleterious outcomes of coronary heart diseases are generally caused by *Qi* (energy) deficiency and obstruction of the coronary circulation (Hou et al., 2014). Therefore, several Chinese herbal remedies with the effects of nourishing *Qi* and activating blood circulation have been widely utilized in ischemic heart disease, including angina pectoris, MI, and heart failure (Mao et al., 2015, 2016b; Li X.Q. et al., 2016). Tongguan capsules (TGC) have received more attention due to their certified cardioprotective effects in human and animal models (Chen et al., 2012; Qi et al., 2013; Ma et al., 2015). Our previous data showed that TGC could markedly decrease cardiac injury biomarkers that led to improved cardiac function in rats with experimental MI (Chen et al., 2012). Most recently, in preclinical myocardial ischemia/reperfusion injury models, the administration of TGC prevented the occurrence of arrhythmias and limited infarction size (Qi et al., 2013). Importantly, the clinical application of TGC had a remarkable therapeutic effect on patients with coronary heart disease, which was evident in the regression of coronary atherosclerosis assessed by coronary computed tomography scans (Ma et al., 2015). These desirable pharmacological effects may be ascribed to the active components of this compound, mainly consisting of *Salvia miltiorrhiza, Hirudo medicinalis*, and *Astragalus membranaceus.* Based on the results of high-performance liquid chromatography fingerprint analysis, this patented medicine has been identified in nearly 15 active chemical components, including salvianolic acid B, fermlononetin, tanshinone I, tanshinone IIA, cryptotanshinone, and salvianolic acid A, most of which are established for cardioprotective therapies (**Figure 1**) (Pan et al., 2011; Zhang et al., 2011; Yuan et al., 2014; Lin et al., 2016; Oche et al., 2016). Inspired by the supportive evidence, we conducted this study to assess the efficacy of TGC on the pathological myocardial remodeling in mice subjected to MI. We demonstrated improved cardiac function and attenuated pathological adverse myocardial remodeling in post-infarct mice treated with TGC. Mechanistically, the protective role of TGC was largely a consequence of its inhibition of apoptosis and provocation of autophagy through silent information regulator 1 (Sirt1).

## Materials and Methods

### Experimental Design

The experimental procedures were performed according to the Guide for the Care and Use of Laboratory Animals published by the US National Academy of Sciences (8th edition, Washington DC, 2011). The Institutional Animal Care and Use Committee of Guangdong Province Hospital of Chinese Medicine at Guangzhou University of Chinese Medicine approved the experimental animal protocol. All reagents were obtained from Sigma-Aldrich Chemicals (St. Louis, MO, United States) unless otherwise specified.

After thoracotomy surgery, mice were randomized into six groups: sham operated mice given vehicle (saline) or TGC; MI mice given vehicle or TGC; MI mice given TGC plus 3MA (autophagy inhibitor); and MI mice given TGC plus EX527 (Sirt1 inhibitor). TGC (batch number 161201; Guangdong Province Hospital, Guangzhou,) was dissolved in 0.9% normal saline and delivered as a daily oral gavage at a dose of 5 mg/kg for 6 weeks. Previous data demonstrated its effect and safety (Qi et al., 2013). The 3MA was administered by intraperitoneal injection (15 mg/kg, twice per week) for 6 weeks. EX527 was intraperitoneally injected at the dose of 5 mg/kg twice per week for 6 weeks. These doses of 3MA and EX527 were previously reported not to cause apparent adverse effects in the rodent heart (Wu et al., 2014; Sin et al., 2015). After 6 weeks, the surviving mice were subjected to echocardiographic assessment; subsequently, the hearts were extracted for histology, enzyme-linked immunosorbent assay (ELISA), western blotting or other analysis.

### Mouse Model of MI

Three-month-old male C57BL/6 mice were obtained from the Experimental Animal Center of Guangdong Province (Guangzhou, China). After being weighed, the mice were anesthetized, intubated, and mechanically ventilated using a rodent ventilator. The hearts were exposed by thoracotomy and subsequent pericardiotomy. MI was induced by permanent left anterior descending coronary artery ligation using 8-0 silk suture, and then confirmed by ST segment elevation or the newly emerging pathological Q waves on an electrocardiogram using the BL-420S Biological Function Experimental System (TME, Shanghai, China). Sham-operated animals underwent the same procedure without ligation of the left coronary artery.

### Transthoracic Echocardiography

Cardiac LV function and structure were non-invasively examined with transthoracic echocardiography via the Vevo 770 high-resolution imaging system (Visual Sonics, Toronto, Canada) using two-dimensional guided M-mode echocardiography, a 15-MHz RMV-707B scanning head before and 3 days and 6 weeks after coronary ligation. All mice were anesthetized using 2% isoflurane/oxygen inhalation before the echocardiography examination. The M-mode measurements were acquired at the level of the papillary muscles with the cursor positioned perpendicular to the interventricular septum and posterior wall. LV dimensions including LV end diastolic dimension (LVEDD) and LV end systolic dimension (LVESD) were averaged from more than 10 cardiac cycles according to the Guidelines of the American Society of Echocardiography (Manning et al., 1994).

### Cardiac Histology

Six weeks after the coronary ligation, the mice were anesthetized and their hearts were harvested, rinsed in normal saline, and surface-dried. The LV myocardium was weighed after removal of the atrial and right ventricular myocardium. The heart mass index (heart mass/body mass in milligrams per gram) were calculated. Subsequently, the ventricular myocardium was flash-frozen in liquid nitrogen and stored at 80°C until used in molecular analyses or fixed in specific fixatives for the histological analyses. The entire procedure was performed in cold conditions.

The murine heart specimens were fixed in 4% formaldehyde overnight and embedded in paraffin. After deparaffinization and rehydratation, 4-μm-thick sections were prepared, mounted on glass slides, and stained with hematoxylin and eosin or Masson’s trichrome staining. Cardiac scar circumference and fibrosis were quantitatively analyzed using a microscope at 40× magnification and a color image analyzer (QWinColour Binary 1; Kirkland, LEICA).

### Terminal Deoxynucleotidyl Transferase dUTP Nick End Labeling Staining

The DNA fragmentations were determined using a Fluorescein-FragEL kit (Oncogene Research Products, Boston, MA, United States) according to the manufacturer’s instructions. Terminal deoxynucleotidyl transferase dUTP nick end labeling (TUNEL) staining was visualized using specific green fluorescence and nuclei by 4′-6-diamidino-2-phenylindole (DAPI). The number of TUNEL-positive cardiomyocytes nuclei was counted and normalized per total nuclei identified by DAPI staining in the same sections using NIS-Element imaging software (Nikon Instruments, Tokyo, Japan).

### Immunofluorescence

After deparaffinization, the sections were incubated with a primary antibody against microtubule-associated LC3 (MBL International, Woburn, MA, United States). To observe autophagic activity in the cardiomyocytes, sections immunostained with anti-LC3 followed by Alexa 568 (red; Molecular Probes, Sunnyvale, CA, United States) were also labeled with anti-myoglobin antibody (DAKO Japan, Kyoto, Japan) followed by Alexa 488 (green; Molecular Probes). These sections were then counterstained with Hoechst 33342 and examined under an Eclipse E1000 microscope and Nikon Digital Sight Camera (Nikon Instruments, Tokyo, Japan).

### Electron Microscopy

Cardiac sample sections from different treatment groups were cut into 1-mm-thick slices and fixed with glutaraldehyde in sodium cacodylate buffer overnight at 37°C. The specimens were washed in distilled water, stained with 2% osmium tetroxide and 0.5% uranyl acetate, dehydrated through a graded series of ethanol and propylene oxide, embedded in epoxy resin, and polymerized for 3 days. Ultrathin sections of 90 nm were cut and mounted on Formvar-coated slot copper grids. Images were acquired of the thin sections using a FEI Tecnai 12 transmission electron microscope (Hitachi H-600, Tokyo, Japan) equipped with a Veleta CCD digital camera (Olympus Soft Imaging Solutions GmbH, Münster, Germany). For quantification, 20 random regions for each sample were considered.

### Enzyme-Linked Immunosorbent Assay

Levels of inflammatory cytokines including tumor necrosis factor (TNF)-α, interleukin (IL)-1β, and IL-6 in the myocardium were measured using commercially available ELISA kits (BioTech, MN, United States). All spectrophotometric readings were obtained with a microplate reader according to the manufacturer’s instructions (Multiskan MK3, Thermo Scientific, United States).

### Western Blotting

Left ventricular samples were homogenized in ice-cold buffer containing 20 mM Tris–HCl, 1 mM EDTA, 5 mM magnesium citrate, 3 mM phenylmethylsulfonyl fluoride, 2 mM sodium orthovanadate, 20 mg/ml aprotinin, and a protease inhibitor cocktail. The prepared samples were analyzed using western blotting as previously described (Mao et al., 2016a). Briefly, 30 μg of protein was loaded into each lane, separated with 8–12% sodium dodecyl sulfate-polyacrylamide gel, and then transferred onto a polyvinylidene difluoride membrane. The membrane was probed first with primary antibodies [anti-GAPDH, anti-ANP, anti-caspase3, anti-Bcl2, anti-Bax, anti-LC3, anti-p62, anti-mammalian target of rapamycin (mTOR), and anti-Sirt1] overnight at 4°C and then with a horseradish peroxidase-conjugated goat anti-rabbit (1:3000) or goat anti-mouse (1:3000) IgG secondary antibody for 2 h at room temperature. The probed protein bands were visualized with enhanced chemiluminescence reaction (Amersham Life Sciences Inc., Marlborough, MA, United States) and were quantified using densitometry (Chemidoc, Biorad, United States). All experiments were separately repeated at least three times.

### Statistical Analysis

Data are presented as the groups and were compared using Student’s *t*-test or one-way analysis of variance using SPSS 12.0 software for Windows. The cumulative survival rate of the rats after MI was analyzed by the Kaplan–Meier method with a log-rank test. Values of *P* < 0.05 were considered significant.

## Results

### TGC Improved Cardiac Function of Mice After Coronary Ligation

**FIGURE 2:**
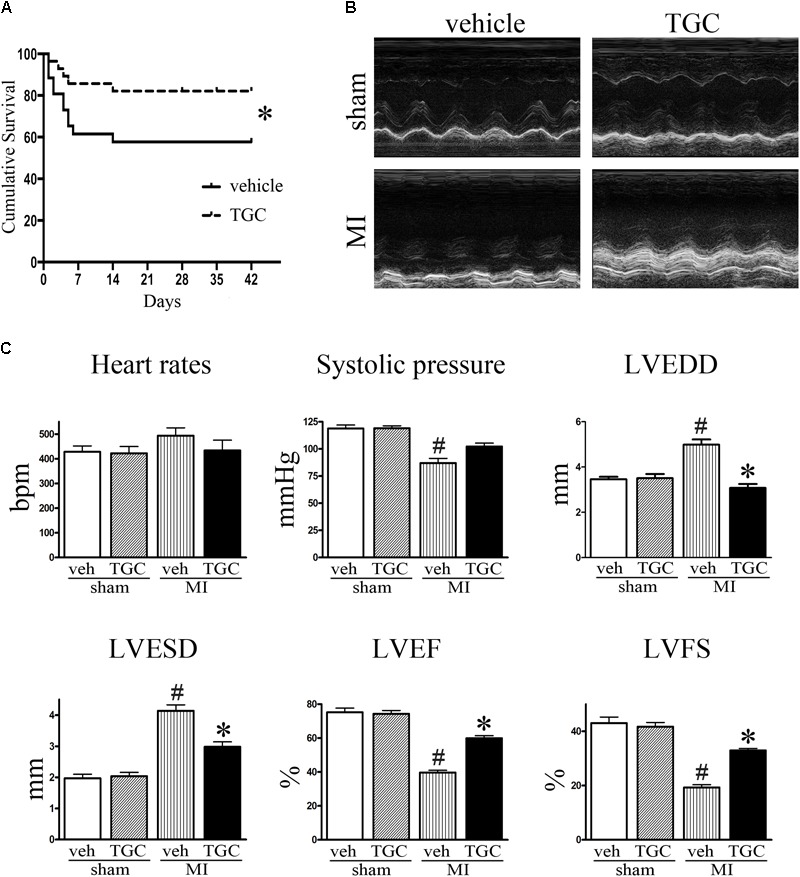
TGC improved mice survival, cardiac morphology and function after MI. **(A)** Kaplan–Meier survival curve of MI mice treated with TGC (*n* = 28) or vehicle (*n* = 26), χ^2^ = 3.94, *P* < 0.05. **(B)** Representative image of echocardiography of mice at 6 weeks after MI. **(C)** Quantitative analysis of LV ejection fraction (LVEF), fractional shortening (LVFS), left ventricular end-diastole diameter (LVEDD), LV end-systolic diameter (LVESD), heart rates, and systolic pressure. ^#^*P* < 0.05 versus sham mice treated with vehicle; ^∗^*P* < 0.05 versus MI mice treated with vehicle. Veh, vehicle; TGC, Tongguan capsules.

During the first 2 days after coronary ligation, the vehicle and TGC groups had similar mortality rates. However, the overall survival rates of the vehicle group were considerably lower than those of TGC group at 42 days after coronary ligation (57.69% vs. 82.14%, *P* < 0.05) with a hazard ratio of 2.77 (95% confidence interval, 1.11–7.57). Furthermore, no mice died in the sham groups treated with TGC or vehicle (**Figure 2A**).

Echocardiography was performed to assess the cardiac function of mice at 3 days and 6 weeks after coronary ligation. Mice from the TGC and vehicle groups showed notable cardiac dysfunctional at 3 days after surgery, without noticeable intergroup differences. However, compared to the vehicle group, the TGC group demonstrated reduced LVEDD and LVESD, improved percent ejection fraction (%EF), and improved fractional shortening (%FS) at 6 weeks after coronary ligation (**Figures 2B,C** and Supplementary Table S1).

Along with echocardiography, hemodynamic analysis suggested a higher systolic pressure in the TGC group than the vehicle group. In the TGC group, there was a trend toward a decreased heart rate compared to the vehicle group, although the statistical difference was not significant. Moreover, we found no significant difference in cardiac function between TGC- and vehicle-treated mice that received sham surgery, suggesting that TGC does not affect LV function under non-surgical conditions.

### TGC Ameliorate Ventricular Fibrosis in Mice After Coronary Ligation

**FIGURE 3:**
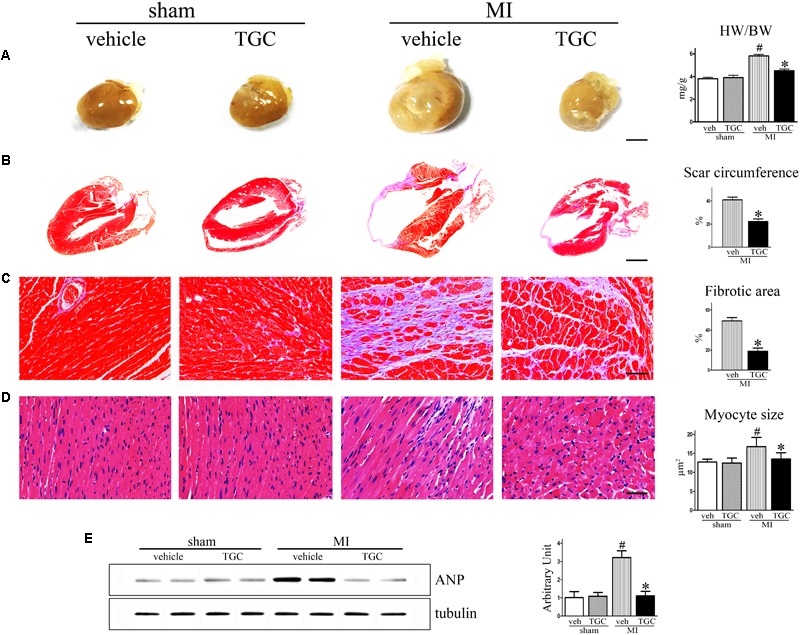
TGC ameliorated ventricular fibrosis after MI. **(A)** Hearts from vehicle-treated mice appear more spherical relative to TGC-treated and sham mice, scale bar = 3 mm. **(B,C)** Paraffin-embedded sections of myocardium were stained with Masson’s trichrome showing the effects of TGC on scar circumference (scale bar = 5 mm) and ventricular fibrosis (scale bar = 50 μm). **(D)** Representative image of hematoxylin and eosin showing myocyte size from mice treated with TGC or vehicle; bars = 50 μm. **(E)** Atrial natriuretic peptide expression in infracted hearts following TGC treatment was assessed by western blotting analysis. Graph right shows densitometric quantification. ^#^*P* < 0.05 versus sham mice treated with vehicle; ^∗^*P* < 0.05 versus MI mice treated with vehicle. Veh, vehicle; TGC, Tongguan capsules.

After MI, the heart undergoes structural remodeling, resulting in a more spherical shape. Isolated hearts from the TGC group demonstrated a less spherical shape than those from the vehicle group 6 weeks after MI, indicating mitigated global cardiac remodeling in mice treated with TGC (**Figure 3A**).

The most striking finding of our study was a marked difference in scar expansion. In vehicle-treated mice, the scar comprised a much larger percent circumference of LV than in the TGC group, indicating the post-MI scar expansion was attenuated by TGC (**Figure 3B**).

Interstitial fibrosis is the hallmark of cardiac remodeling (Mao et al., 2014). As shown in **Figure 3C**, fibrotic areas were reduced in the TGC heart, suggesting suppressed interstitial fibrosis in the TGC-treated heart.

Myocyte size were analyzed in the border and the remote zones (**Figure 3D**). Increased myofilament density and decreased myocyte size were observed in the border zone of the TGC group compared to the vehicle group. In contrast, no significant difference in either myofilament density or myocyte size was observed in the remote zone between the two groups, suggesting that TGC attenuated myocyte hypertrophy and secondary cell loss in the border zone after MI.

It has been established that atrial natriuretic peptide (ANP) closely correlates with cardiac remodeling and dysfunction severity (Omland et al., 1996). Therefore, we assessed ANP expression in vehicle and TGC mice using western blotting analysis. As revealed in **Figure 3E**, ANP expression level was significantly downregulated in the TGC group compared to the vehicle group, suggesting the attenuation of deteriorated LV function in the TG group. Collectively, these results reveal that TGC treatment improved cardiac remodeling after MI.

### TGC Decreased Inflammatory Cytokine Expression in Mice Subjected to Coronary Ligation

**FIGURE 4:**
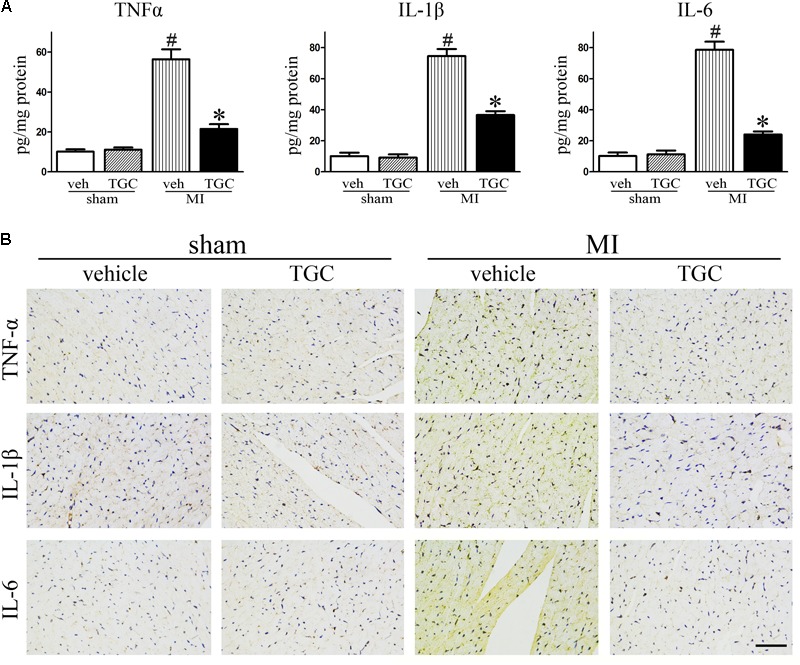
TGC decreased inflammatory factors expression after MI. **(A)** The production of TNF-α, IL-1β, and IL-6 assessed by ELISA kits in the myocardium. **(B)** Immunohistochemical analysis of inflammatory factors expression in the infarct border zone of the heart (scale bar = 50 μm). ^#^*P* < 0.05 versus sham mice treated with vehicle; ^∗^*P* < 0.05 versus MI mice treated with vehicle. Veh, vehicle; TGC, Tongguan capsules.

Myocardial ischemia initiates an inflammatory response characterized by an accumulation of leukocytes in the injured myocardium, while cytokines expression further promotes adverse LV remodeling and heart failure (Velagaleti et al., 2008). To elucidate the effect of TGC on post-infarct inflammation, we evaluated the expression of TNF-α, IL-1β and IL-6 in the myocardium using commercial ELISA kits. TGC dramatically decreased the expressions of TNF-α, IL-1β, and IL-6 in the myocardium tissue compared to the vehicle group (**Figure 4A**).

In parallel, there was a remarkable decrease in immune-detectable antibodies against inflammatory factors TNF-α, IL-1β, and IL-6 in the presence of TGC in the myocardium, which further supports results obtained from ELISA (**Figure 4B**).

### TGC Inhibited Apoptosis in Mice After Coronary Ligation

**FIGURE 5:**
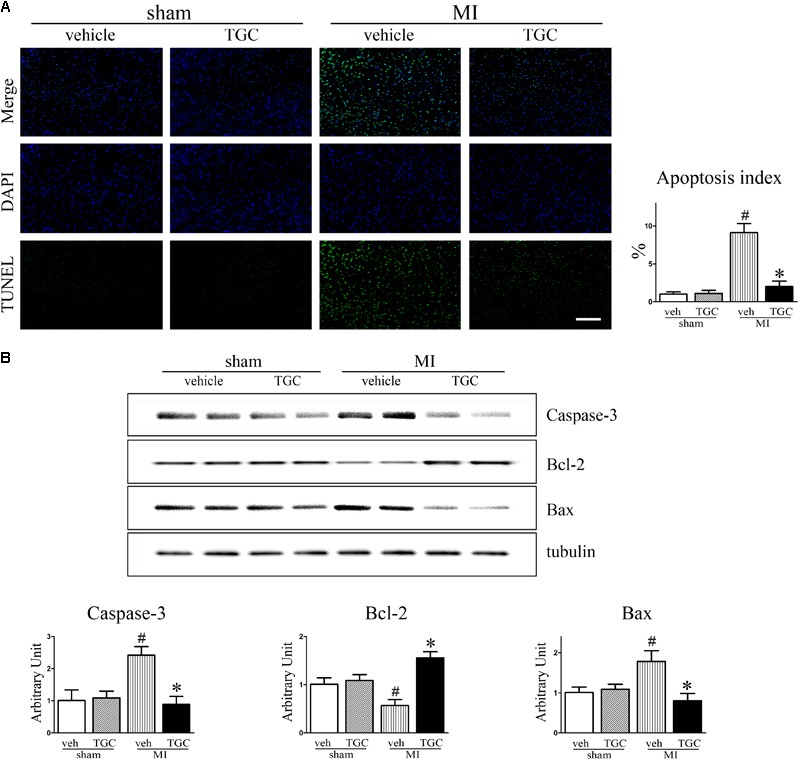
TGC attenuated apoptosis after MI. **(A)** Apoptosis in myocardium from TGC or vehicle treated mice and sham group was evaluated by TUNEL assay. TUNEL-positive cells were significantly reduced in TGC group compared to the vehicle group, whereas few TUNEL-positive cells were detectable in the sham group (scale bar = 100 μm). DAPI nuclear staining in blue. **(B)** The caspase 3, Bcl-2, and Bax levels were assessed by western blotting analysis. Graph below shows densitometric quantification (*n* = 4 per group). ^#^*P* < 0.05 versus sham mice treated with vehicle; ^∗^*P* < 0.05 versus MI mice treated with vehicle. Veh, vehicle; TGC, Tongguan capsules.

Cardiomyocyte apoptosis contributes to post-infarct cardiac remodeling and subsequent cardiac dysfunction (Haudek et al., 2007). To identify whether TGC protects the post-infarct myocardium against apoptosis, we assessed apoptotic biomarkers expression using TUNEL staining and western blotting analysis. There were dramatically fewer apoptotic (TUNEL-positive) cells on the border of the MI hearts of the TGC group compared to the vehicle group (**Figure 5A**), suggesting that TGC anti-apoptotic properties. This result was confirmed by measurement of the cleaved caspase 3, B cell lymphoma-2 (Bcl-2), and Bcl-2-associated X (Bax) via western blotting analysis. There was a significant increase in Bcl-2 and decrease in cleaved caspase 3 and Bax at the protein level on the border of the MI hearts after TGC treatment (**Figure 5B**).

### Upregulation of Autophagy by TGC in Mice After Coronary Ligation

In supporting evidence, autophagy, associated with cardiomyocytes survival in ischemia, is involved in the regulation of LV remodeling; thus, we investigated whether TGC attenuated LV remodeling by regulating cell autophagy after MI.

**FIGURE 6:**
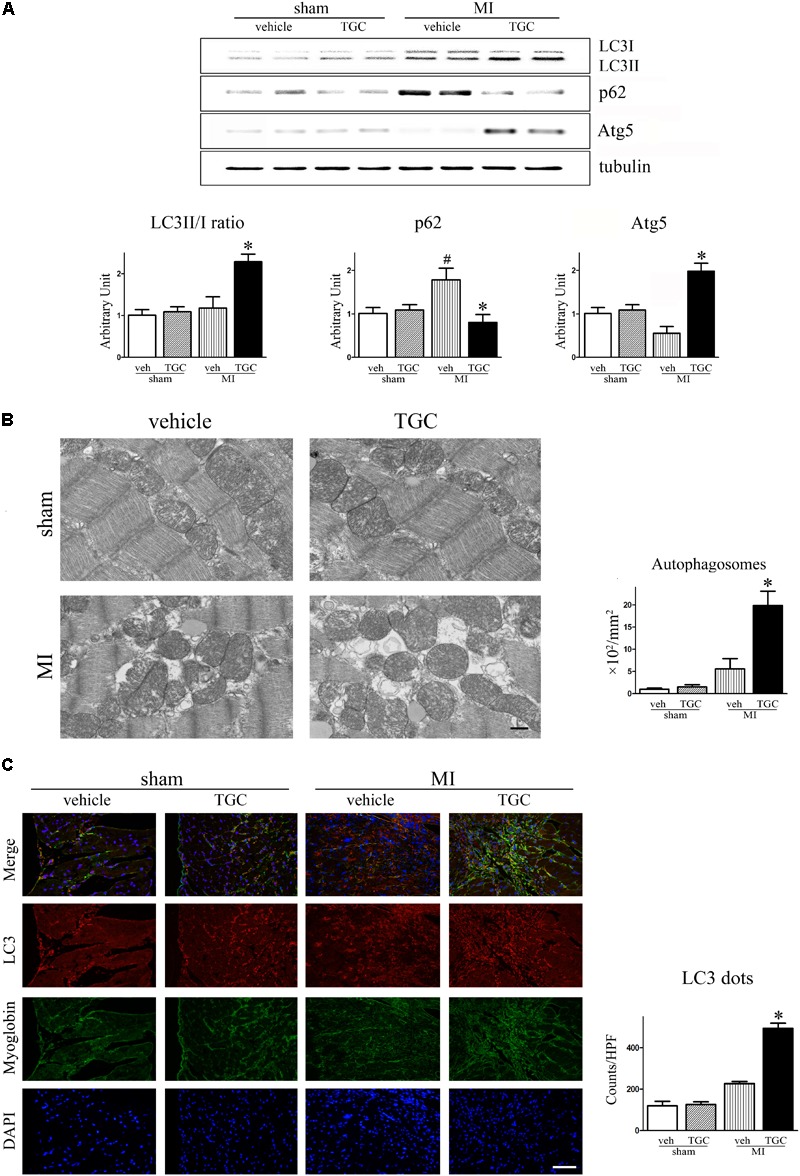
TGC regulated autophagy after MI. **(A)** The expression of LC3II/LC3I, p62, and Atg5 in infracted heart was assessed by western blotting analysis. **(B)** Electron micrographs show more autophagic vacuole formation in myocyte of MI mice treated with TGC. Quantification of autophagy by measuring the average number of autophagic vacuoles per 10000^∗^ filed in electron microscope images; scale bars = 500 nm. **(C)** Representative images of LC3 dots LV tissue sections of post-MI heart; red, LC3; blue, DAPI-stained nuclei; green, myoglobin-positive cardiomyocytes; original magnification ×400 (scale bar = 50 μm). ^#^*P* < 0.05 versus sham mice treated with vehicle; ^∗^*P* < 0.05 versus MI mice treated with vehicle. Veh, vehicle; TGC, Tongguan capsules.

The LC3-II to LC3-I ratio and Atg5 level were used to assess autophagy in the post-infarct myocardium, while the autophagy substrate SQSTM1/p62 was associated with autophagic flux (Klionsky et al., 2016). There was an upregulation of the autophagic molecular marker, Atg5, in the TGC group compared to the vehicle group (**Figure 6A**). Furthermore, LC3-II to LC3-I ratio was increased but the SQSTM1/p62 expression was decreased in the TGC-treated MI hearts, suggesting that TGC causes enhanced autophagy in cardiomyocytes in response to myocardial ischemia and infarction. The enhanced autophagy in TGC-treated MI hearts was further confirmed by the increased autophagic vacuoles on electron microscopy (**Figure 6B**) and LC3-positive punctate staining assessed by immunofluorescence (**Figure 6C**).

### Inhibition of Autophagy Attenuated TGC-Induced Anti-Remodeling Effect

**FIGURE 7:**
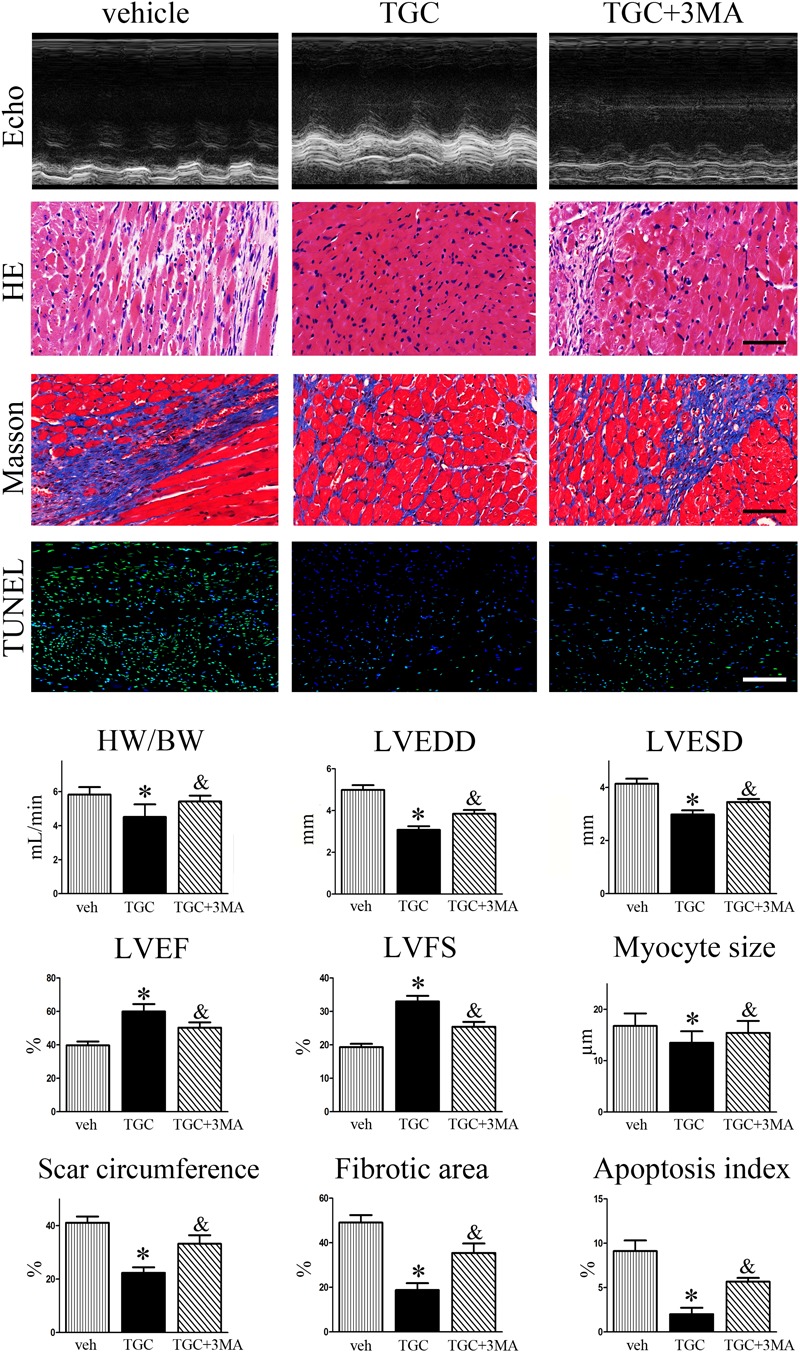
Inhibition of TGC-induced autophagy enhanced LV remodeling after MI. MI mice were treated with TGC in the absence or presence of 3MA. Representative pictures of echocardiography, hematoxylin, and eosin (scale bar = 50 μm), Masson’s trichrome (scale bar = 50 μm), and TUNEL (scale bar = 100 μm) stained LV tissue sections are shown in the upper panels. Quantitative analysis of heart weight to body weight ratio, left ventricular end-diastole diameter (LVEDD); LV end-systolic diameter (LVESD), LV ejection fraction (LVEF) and fractional shortening (LVFS), infarct size, fibrosis area, and apoptosis index are shown in lower panels. ^∗^*P* < 0.05 versus MI mice treated with vehicle, ^&^*P* < 0.05 versus MI mice treated with TGC. Veh, vehicle; TGC, Tongguan capsules.

Considering that accumulating evidence suggests that autophagy plays dual roles in cytoprotection and cell death, we then evaluated the effects of autophagy in the TGC-induced anti-remodeling effect in post-infarct hearts. As shown in **Figure 7**, combination treatment with autophagic inhibitor 3MA resulted in a significantly increased ratio of heart weight to body weight, dilatation of LV-internal dimension, scar circumference, fibrosis area, and decrease of LVEF or FS compared to TGC treatment alone. Furthermore, TUNEL staining also demonstrated that combination treatment of 3MA diminished TGC-induced anti-apoptosis effects (**Figure 7**). Taken together, the results indicated that TGC-induced autophagy plays an important protective role against cardiac injury and apoptosis after MI.

### TGC Downregulated the mTOR/P70/S6K/4EBP1 Pathway in Mice After Coronary Ligation

**FIGURE 8:**
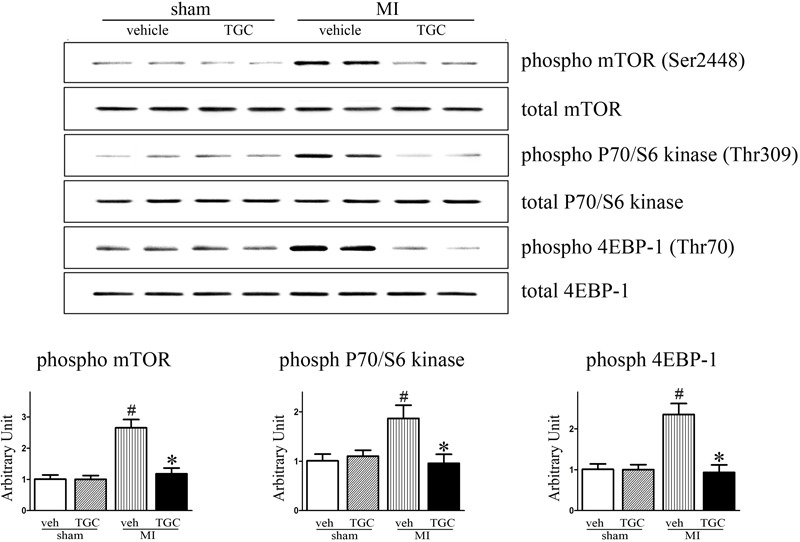
TGC negatively regulated mTOR/P70/S6 kinase signaling pathway. Phosphorylation of mTOR (Ser2448) and other known downstream molecular targets including P70/S6- kinase (Thr309) and 4EBP1 (Thr70) in the hearts of MI mice treated with TGC or vehicle assessed by western blotting analysis. ^#^*P* < 0.05 versus sham mice treated with vehicle; ^∗^*P* < 0.05 versus MI mice treated with vehicle. Veh, vehicle; TGC, Tongguan capsules.

Existing data demonstrate that mTOR and its downstream effectors P70S6K and 4EBP1 are involved in autophagy after ischemia (Guo et al., 2012). Accordingly, we determined whether TGC enhanced autophagy in MI mice via regulation of the mTOR/P70/S6K/4EBP1 pathway in the MI heart. First, we examined the mTOR expression by western blot analysis. As shown in **Figure 8**, TGC markedly reduced the mTOR (Ser2448) phosphorylation levels. Next, we examined the phosphorylation state of P70S6K (Thr309) and 4EBP1 (Thr70), established targets of the mTOR1 complex. As shown in **Figure 8**, TGC also significantly decreased the phosphorylation levels of P70S6 kinase and 4EBP1. These results revealed that TGC is an effective inhibiter of the mTOR pathway in post-MI hearts.

### TGC Induced Autophagy and Decreased Apoptosis Through Sirt1 Activation

**FIGURE 9:**
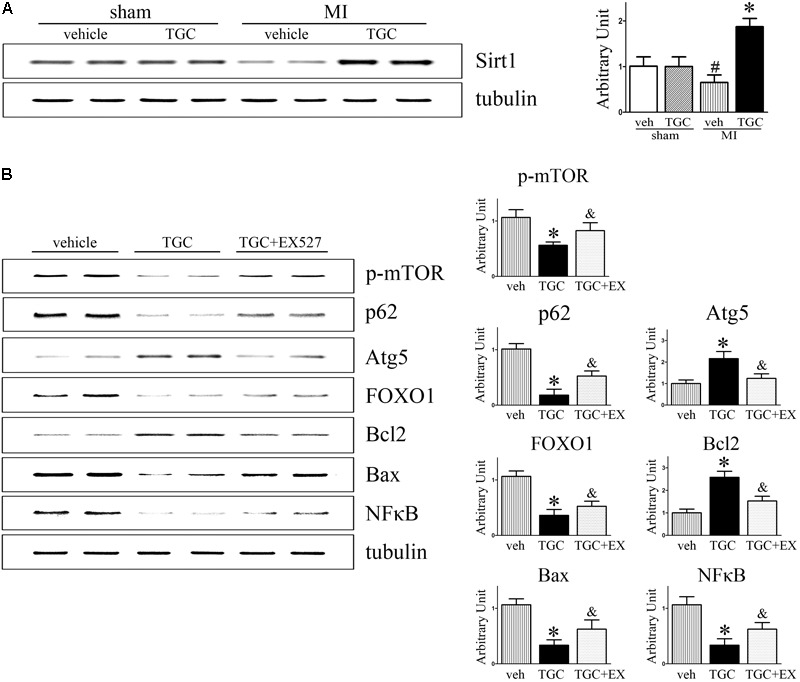
Inhibition of Sirt1 suppressed TGC induced autophagy while increasing apoptosis in mice with MI. **(A)** Immunoblot analyses of Sirt1 in the heart homogenates of MI mice treated with TGC or vehicle. **(B)** MI mice were treated with TGC alone or TGC plus Sirt1 inhibitor EX527. The representative immunoblot analysis images of phosphorylation of mTOR (Ser2448), p62, Atg5, FOXO1, Bcl-2, Bax, and NFκB are shown. ^∗^*P* < 0.05 versus MI mice treated with vehicle, ^&^*P* < 0.05 versus MI mice treated with TGC. Veh, vehicle; TGC, Tongguan capsules.

It was previously reported that silent information regulator 1 (Sirt1) induced autophagy by inhibiting mTOR signaling (Salminen and Kaarniranta, 2009). For this reason, we next evaluated whether Sirt1 is involved in TGC-induced autophagy in mice with coronary ligation. As shown in **Figure 9**, TGC treatment indeed promoted Sirt1 expression in post-infarct hearts compared to vehicle treatment.

As a NAD-dependent deacetylase, Sirt1 was demonstrated to regulate autophagy via its enzymatic activity (Lee et al., 2008). As expected, EX-527, a specific inhibitor of Sirt1 activity, attenuated TGC-induced autophagy as evidenced by decreased Atg5 levels and rescue of p62 and phosphorylated mTOR (Ser2448) levels (**Figure 9B**). Collectively, these results indicated that TGC-induced autophagy is mediated by Sirt1 activation and subsequently downregulates mTOR pathway mediators.

Moreover, the inhibition of Sirt1 activity by EX-527 increased forkhead box O1 (FOXO1) and Bax expressions and markedly decreased Bcl2 and NFκB expression compared to those in the TGC group (**Figure 9B**). These data suggest that TGC inhibits apoptosis and inflammation via Sirt1 as well.

## Discussion

Cardiac remodeling and dysfunction is a determinant of heart failure progression following MI. During the response to ischemic injury, in the ischemic and the remote non-infarcted myocardium, progressive LV remodeling occurs, which includes cardiomyocytes apoptosis, hypertrophy, and fibrosis (Bhatt et al., 2017). Developing strategies to reduce post-infarct cardiac remodeling has become a major therapeutic challenge. In the present study, we confirmed a novel therapeutic approach to the treatment of post-MI remodeling using TCM. Our results suggest that TGC significantly attenuated pathological LV remodeling attributed to enhanced autophagy, reduced apoptosis and inflammation, and upregulated Sirt1 activation. Furthermore, a specific Sirt1 inhibitor abolished the pro-autophagy, anti-apoptotic, and anti-inflammatory effect of TGC as evidenced by the increased p62, Bax, and NFκB expressions and reduced Atg5 and Bcl-2 expressions. Thus, these results indicate that Sirt1 activation by TGC contributes to the attenuated cardiac remodeling after MI.

Sirt1 has drawn much attention for its ability to mediate longevity stimulated by resveratrol, a component of red wine seeds (Howitz et al., 2003). Importantly, Sirt1 is thought to play a crucial role in cardioprotection against myocardial ischemia/reperfusion injury through sumoylation which in turn induces lysine deacetylation of cytosolic proteins (Nadtochiy et al., 2011a). Sirt1 also mediates the protective effect by decreasing the transcriptional activity of NFκB through deacetylation of its subunit p65, which may in turn prevent inflammation and consequent cardiac remodeling (Nadtochiy et al., 2011b). Consistent with these findings, we found that TGC augmented Sirt1 expression as well as inhibited pro-inflammatory factors.

Several studies have shown that Sirt 1 activity inhibits apoptosis by deacetylating p53, a known apoptosis facilitator (Yamamoto and Sadoshima, 2011). Sirt1 downregulation may contribute to rapid p53 activation and the consequent apoptotic death of cardiomyocytes during ischemia (Hsu et al., 2010). In addition, Sirt1 overexpression in transgenic mice induced deacetylation and nuclear translocation of FoxO1. This subsequently led to the upregulation Bcl-XL and Bcl-2 upregulation and Bax downregulation (Hsu et al., 2010). This study also demonstrated that the specific inhibition of Sirt1 activity abolished TGC-induced anti-apoptotic effects. Accordingly, declined myocardial apoptosis via Sirt1 is one mechanisms by which TGC attenuated post-MI cardiac remodeling. However, the precise mechanisms by which TGC-activated Sirt1 controls gene expression through FoxO1 during the pathological remodeling process remain to be elucidated.

Autophagy is normally an important homeostatic mechanism that involves degradation and recycling of cytoplasmic components and organelles for maintaining myocyte function (Lum et al., 2005). In cardiac remodeling, an adaptive response protects cardiomyocytes against ischemic and hemodynamic stress by eliminating misfolded or unfavorably modified proteins and dysfunctional organelles that could disrupt cardiac function (Ceylan-Isik et al., 2013; Thomas et al., 2013). Recent studies have verified that Sirt1 activation could induce autophagy in cardiomyocytes and attenuate cardiac remodeling and contractile dysfunction (Zhang et al., 2014). In the absence of autophagy, the accumulation of polyubiquitinated proteins may be responsible for extended endoplasmic reticulum stress, resulting in apoptosis (Nakai et al., 2007). Moreover, a previous study demonstrated that the increased cardiomyocyte autophagy acted as a cardioprotective response against apoptosis under a glucose deprivation condition through AMPK activation and mTOR inhibition (Matsui et al., 2007). Here we found that the LC3-II/LC3-I ratio and autophagic vacuoles were significantly higher in TGC-treated mice than in vehicle-treated mice. However, the exact mechanism by which autophagy of cardiomyocytes protects against apoptosis in post-infarct hearts is unclear. Increasing evidence indicates that a potential mechanism is associated with the crosstalk between the autophagy initiation protein Beclin1 (BECN1) and anti-apoptotic protein Bcl-2, a switch between autophagy and apoptosis. Studies have demonstrated a strong link between Bcl-2 and BECN1 accompanied by the inhibition of autophagy in post-MI hearts with cardiac remodeling (Maejima et al., 2013). In contrast, disruption of Bcl-2 and BECN1 interaction gives rise to BECN1 being bound by the class III PtdIns3K to initiate autophagy (Maejima et al., 2016). In addition, phosphorylated Bcl-2 could stabilize the mitochondrial outer membrane and prevent pro-apoptotic proteins such as cytochrome C from escaping (or being released) into the cytoplasm (Yang et al., 1997). There are several other potential factors that regulate both autophagy and apoptosis, including Atgs, caspases, p53, and FADD-like IL-1β-converting enzyme-inhibitory protein (FLIP) but further studies are needed to decipher the mechanism by which TGC regulates the switch between autophagy and apoptosis after MI (Li M. et al., 2016).

Autophagy has been demonstrated to play a dual role in cardiomyocyte survival. Both profitable and detrimental effects of autophagy have been described when cardiomyocytes were exposed to various stimuli (Gustafsson and Gottlieb, 2008). Therefore, it remains to be elucidated whether TGC-induced autophagy in the post-infarct myocardium is required for survival and thus was salutary or mediated the cell death and is thereby detrimental. In this study, the inhibition of TGC-induced autophagy by 3MA was accompanied by decreased cardiac dysfunction and cardiomyocyte survival, collectively suggesting that TGC-induced autophagy was protective in these processes. We deduced that the improved cardiac LV function was associated with enhanced autophagy, which facilitated animals to better respond to adverse ischemic stress (Matsui et al., 2007). The potential role of autophagy as a therapeutic target should be considered in future studies, while the mechanism of TGC-induced autophagy should be illustrated in detail.

The mTOR and its downstream signaling pathways are key regulators of autophagy and involved in pathological cardiac remodeling and even heart failure (Buss et al., 2009). However, the role of mTOR in ischemic heart disease remains undefined. Existing data shows that cardiac genetic mTOR overexpression was sufficient to provide substantial cardioprotection against ischemia-reperfusion injury both *in vivo* with transient coronary artery ligation and in Langendorff-perfused hearts with transient global ischemia (Aoyagi et al., 2012). However, mTOR inhibition has also been shown to have definite protective effects when the myocardium suffers from ischemic or another stress stimulus by promoting autophagy (Shioi et al., 2003). Previous studies demonstrated that activated mTORC1 is associated with pathological cardiac remodeling in the heart (Ozcan et al., 2008). In contrast to Shioi’s reports, we demonstrated here that TGC significantly inhibited the mTOR pathway, leading to increased autophagy in the context of myocardial ischemia. Considering that the mTOR plays a critical role in the cross-talk between autophagy and apoptosis, it is necessary to further investigate how the mTOR signaling pathway precisely regulates the switch between autophagy and apoptosis in TGC-treated cardiomyocytes (Eisenberg-Lerner et al., 2009).

In conclusion, our study demonstrated that TGC improved post-MI cardiac remodeling by affecting SIRT1 activity and subsequently enhancing autophagy, inhibiting apoptosis, and inflammation. These factors contribute to the cardioprotective role in post-MI. Therefore, TGC serves as a promising therapeutic candidate for the treatment for ischemic heart disease.

## Author Contributions

SM drafted this manuscript. SM, PC, and TL performed the experiments. LG did the statistical analysis. MZ critically revised the manuscript and contributed to the rationalization of the study. All authors read and approved the final manuscript.

## Conflict of Interest Statement

The authors declare that the research was conducted in the absence of any commercial or financial relationships that could be construed as a potential conflict of interest.

## References

[B1] Aki T. Yamaguchi K. Fujimiya T. Mizukami Y. (2003). Phosphoinositide 3-kinase accelerates autophagic cell death during glucose deprivation in the rat cardiomyocyte-derived cell line H9c2. 22 8529–8535. 10.1038/sj.onc.1207197 14627994

[B2] Aoyagi T. Kusakari Y. Xiao C. Y. Inouye B. T. Takahashi M. Scherrer-Crosbie M. (2012). Cardiac mTOR protects the heart against ischemia-reperfusion injury. 303 H75–H85. 10.1152/ajpheart.00241.2012 22561297 PMC3404649

[B3] Bharti S. Rani N. Bhatia J. Arya D. S. (2015). 5-HT2B receptor blockade attenuates beta-adrenergic receptor-stimulated myocardial remodeling in rats via inhibiting apoptosis: role of MAPKs and HSPs. 20 455–465. 10.1007/s10495-014-1083-z 25544272

[B4] Bhatt A. S. Ambrosy A. P. Velazquez E. J. (2017). Adverse remodeling and reverse remodeling after myocardial infarction. 19:71. 10.1007/s11886-017-0876-4 28660552

[B5] Buss S. J. Muenz S. Riffel J. H. Malekar P. Hagenmueller M. Weiss C. S. (2009). Beneficial effects of Mammalian target of rapamycin inhibition on left ventricular remodeling after myocardial infarction. 54 2435–2446. 10.1016/j.jacc.2009.08.031 20082935

[B6] Ceylan-Isik A. F. Dong M. Zhang Y. Dong F. Turdi S. Nair S. (2013). Cardiomyocyte-specific deletion of endothelin receptor A rescues aging-associated cardiac hypertrophy and contractile dysfunction: role of autophagy. 108:335. 10.1007/s00395-013-0335-3 23381122 PMC3590116

[B7] Chen Q. F. Zhang M. Z. Yang C. (2012). [Experimental study of the myocardial protection on septic rats by tongguan capsule]. 32 1253–1257. 23185770

[B8] Cuenin L. Lamoureux S. Schaaf M. Bochaton T. Monassier J. P. Claeys M. J. (2017). Incidence and significance of spontaneous ST segment re-elevation after reperfused anterior acute myocardial infarction- relationship with infarct size, adverse remodeling, and events at 1 year. 82 1379–1386. 10.1253/circj.CJ-17-0671 28943533

[B9] Eisenberg-Lerner A. Bialik S. Simon H. U. Kimchi A. (2009). Life and death partners: apoptosis, autophagy and the cross-talk between them. 16 966–975. 10.1038/cdd.2009.33 19325568

[B10] Guo R. Hu N. Kandadi M. R. Ren J. (2012). Facilitated ethanol metabolism promotes cardiomyocyte contractile dysfunction through autophagy in murine hearts. 8 593–608. 10.4161/auto.18997 22441020 PMC3405837

[B11] Gustafsson A. B. Gottlieb R. A. (2008). Recycle or die: the role of autophagy in cardioprotection. 44 654–661. 10.1016/j.yjmcc.2008.01.010 18353358 PMC2423346

[B12] Hassell M. E. Vlastra W. Robbers L. Hirsch A. Nijveldt R. Tijssen J. G. (2017). Long-term left ventricular remodelling after revascularisation for ST-segment elevation myocardial infarction as assessed by cardiac magnetic resonance imaging. 4:e000569. 10.1136/openhrt-2016-000569 28861274 PMC5577529

[B13] Haudek S. B. Taffet G. E. Schneider M. D. Mann D. L. (2007). TNF provokes cardiomyocyte apoptosis and cardiac remodeling through activation of multiple cell death pathways. 117 2692–2701. 10.1172/JCI29134 17694177 PMC1937497

[B14] Hou J. Wang J. Lin C. Fu J. Ren J. Li L. (2014). Circulating microRNA profiles differ between Qi-stagnation and Qi-deficiency in coronary heart disease patients with blood stasis syndrome. 2014:926962. 10.1155/2014/926962 25548593 PMC4273468

[B15] Howitz K. T. Bitterman K. J. Cohen H. Y. Lamming D. W. Lavu S. Wood J. G. (2003). Small molecule activators of sirtuins extend Saccharomyces cerevisiae lifespan. 425 191–196. 10.1038/nature01960 12939617

[B16] Hsu C. P. Zhai P. Yamamoto T. Maejima Y. Matsushima S. Hariharan N. (2010). Silent information regulator 1 protects the heart from ischemia/reperfusion. 122 2170–2182. 10.1161/CIRCULATIONAHA.110.958033 21060073 PMC3003297

[B17] Klionsky D. J. Abdelmohsen K. Abe A. Abedin M. J. Abeliovich H. Acevedo Arozena A. (2016). Guidelines for the use and interpretation of assays for monitoring autophagy (3rd edition). 12 1–222. 10.1080/15548627.2015.1100356 26799652 PMC4835977

[B18] Kunapuli S. Rosanio S. Schwarz E. R. (2006). ”How do cardiomyocytes die?” apoptosis and autophagic cell death in cardiac myocytes. 12 381–391. 10.1016/j.cardfail.2006.02.002 16762802

[B19] Lee I. H. Cao L. Mostoslavsky R. Lombard D. B. Liu J. Bruns N. E. (2008). A role for the NAD-dependent deacetylase Sirt1 in the regulation of autophagy. 105 3374–3379. 10.1073/pnas.0712145105 18296641 PMC2265142

[B20] Levine B. Klionsky D. J. (2004). Development by self-digestion: molecular mechanisms and biological functions of autophagy. 6 463–477. 10.1016/S1534-5807(04)00099-1 15068787

[B21] Li M. Gao P. Zhang J. (2016). Crosstalk between autophagy and apoptosis: potential and emerging therapeutic targets for cardiac diseases. 17:332. 10.3390/ijms17030332 26950124 PMC4813194

[B22] Li X. Q. He J. C. Huang P. X. Cao X. B. (2016). Chinese medicine syndromes in congestive heart failure: a literature study and retrospective analysis of clinical cases. 22 738–744. 10.1007/s11655-015-2085-6 26906719

[B23] Lin C. Liu Z. Lu Y. Yao Y. Zhang Y. Ma Z. (2016). Cardioprotective effect of Salvianolic acid B on acute myocardial infarction by promoting autophagy and neovascularization and inhibiting apoptosis. 68 941–952. 10.1111/jphp.12567 27139338

[B24] Lum J. J. DeBerardinis R. J. Thompson C. B. (2005). Autophagy in metazoans: cell survival in the land of plenty. 6 439–448. 10.1038/nrm1660 15928708

[B25] Ma H. Wang L. Huang D. Liu G. Zhang M. (2015). Tongguan capsule ameliorates coronary artery stenosis in a 40-year-old woman. 9 4413–4416. 10.2147/DDDT.S85571 26309395 PMC4539080

[B26] Maejima Y. Isobe M. Sadoshima J. (2016). Regulation of autophagy by Beclin 1 in the heart. 95 19–25. 10.1016/j.yjmcc.2015.10.032 26546165 PMC4861696

[B27] Maejima Y. Kyoi S. Zhai P. Liu T. Li H. Ivessa A. (2013). Mst1 inhibits autophagy by promoting the interaction between Beclin1 and Bcl-2. 19 1478–1488. 10.1038/nm.3322 24141421 PMC3823824

[B28] Manning W. J. Wei J. Y. Katz S. E. Litwin S. E. Douglas P. S. (1994). In vivo assessment of LV mass in mice using high-frequency cardiac ultrasound: necropsy validation. 266 H1672–H1675. 10.1152/ajpheart.1994.266.4.H1672 8184946

[B29] Mao S. Li W. Qa’aty N. Vincent M. Zhang M. Hinek A. (2016a). Tanshinone IIA inhibits angiotensin II induced extracellular matrix remodeling in human cardiac fibroblasts–Implications for treatment of pathologic cardiac remodeling. 202 110–117. 10.1016/j.ijcard.2015.08.191 26408838

[B30] Mao S. Li X. Wang L. Yang P. C. Zhang M. (2015). Rationale and design of sodium tanshinone IIA sulfonate in left ventricular remodeling secondary to acute myocardial infarction (STAMP-REMODELING) trial: a randomized controlled study. 29 535–542. 10.1007/s10557-015-6625-2 26482376

[B31] Mao S. Wang L. Ouyang W. Zhou Y. Qi J. Guo L. (2016b). Traditional Chinese medicine, Danlou tablets alleviate adverse left ventricular remodeling after myocardial infarction: results of a double-blind, randomized, placebo-controlled, pilot study. 16:447. 10.1186/s12906-016-1406-4 27825334 PMC5101662

[B32] Mao S. Wang Y. Zhang M. Hinek A. (2014). Phytoestrogen, tanshinone IIA diminishes collagen deposition and stimulates new elastogenesis in cultures of human cardiac fibroblasts. 323 189–197. 10.1016/j.yexcr.2014.02.001 24525372

[B33] Matsui Y. Takagi H. Qu X. Abdellatif M. Sakoda H. Asano T. (2007). Distinct roles of autophagy in the heart during ischemia and reperfusion: roles of AMP-activated protein kinase and Beclin 1 in mediating autophagy. 100 914–922. 10.1161/01.RES.0000261924.76669.36 17332429

[B34] Nadtochiy S. M. Redman E. Rahman I. Brookes P. S. (2011a). Lysine deacetylation in ischaemic preconditioning: the role of SIRT1. 89 643–649. 10.1093/cvr/cvq287 20823277 PMC3028968

[B35] Nadtochiy S. M. Yao H. McBurney M. W. Gu W. Guarente L. Rahman I. (2011b). SIRT1-mediated acute cardioprotection. 301 H1506–H1512. 10.1152/ajpheart.00587.2011 21856913 PMC3197366

[B36] Nakai A. Yamaguchi O. Takeda T. Higuchi Y. Hikoso S. Taniike M. (2007). The role of autophagy in cardiomyocytes in the basal state and in response to hemodynamic stress. 13 619–624. 10.1038/nm1574 17450150

[B37] Oche B. Chen L. Ma Y. K. Yang Y. Li C. X. Geng X. (2016). Cryptotanshinone and wogonin up-regulate eNOS in vascular endothelial cells via ERalpha and down-regulate iNOS in LPS stimulated vascular smooth muscle cells via ERbeta. 39 249–258. 10.1007/s12272-015-0671-y 26481132

[B38] Omland T. Aakvaag A. Bonarjee V. V. Caidahl K. Lie R. T. Nilsen D. W. (1996). Plasma brain natriuretic peptide as an indicator of left ventricular systolic function and long-term survival after acute myocardial infarction. Comparison with plasma atrial natriuretic peptide and N-terminal proatrial natriuretic peptide. 93 1963–1969. 10.1161/01.CIR.93.11.1963 8640969

[B39] Ozcan U. Ozcan L. Yilmaz E. Duvel K. Sahin M. Manning B. D. (2008). Loss of the tuberous sclerosis complex tumor suppressors triggers the unfolded protein response to regulate insulin signaling and apoptosis. 29 541–551. 10.1016/j.molcel.2007.12.023 18342602 PMC2361721

[B40] Pan H. Li D. Fang F. Chen D. Qi L. Zhang R. (2011). Salvianolic acid A demonstrates cardioprotective effects in rat hearts and cardiomyocytes after ischemia/reperfusion injury. 58 535–542. 10.1097/FJC.0b013e31822de355 21795988

[B41] Qi J. Yu J. Wang L. Guo L. Ma S. Huang D. (2013). Tongguan capsule protects against myocardial ischemia and reperfusion injury in mice. 2013:159237. 10.1155/2013/159237 24073004 PMC3774060

[B42] Riquelme J. A. Chavez M. N. Mondaca-Ruff D. Bustamante M. Vicencio J. M. Quest A. F. (2016). Therapeutic targeting of autophagy in myocardial infarction and heart failure. 14 1007–1019. 10.1080/14779072.2016.1202760 27308848

[B43] Salminen A. Kaarniranta K. (2009). SIRT1: regulation of longevity via autophagy. 21 1356–1360. 10.1016/j.cellsig.2009.02.014 19249351

[B44] Sharp T. E. Schena G. J. Hobby A. R. Starosta T. Berretta R. M. Wallner M. (2017). Cortical bone stem cell therapy preserves cardiac structure and function after myocardial infarction. 121 1263–1278. 10.1161/CIRCRESAHA.117.311174 28912121 PMC5681384

[B45] Shioi T. McMullen J. R. Tarnavski O. Converso K. Sherwood M. C. Manning W. J. (2003). Rapamycin attenuates load-induced cardiac hypertrophy in mice. 107 1664–1670. 10.1161/01.CIR.0000057979.36322.88 12668503

[B46] Shirakabe A. Ikeda Y. Sciarretta S. Zablocki D. K. Sadoshima J. (2016). Aging and autophagy in the heart. 118 1563–1576. 10.1161/CIRCRESAHA.116.307474 27174950 PMC4869999

[B47] Sin T. K. Tam B. T. Yung B. Y. Yip S. P. Chan L. W. Wong C. S. (2015). Resveratrol protects against doxorubicin-induced cardiotoxicity in aged hearts through the SIRT1-USP7 axis. 593 1887–1899. 10.1113/jphysiol.2014.270101 PMC4405749 25665036

[B48] Thapalia B. A. Zhou Z. Lin X. (2014). Autophagy, a process within reperfusion injury: an update. 7 8322–8341. 25674198 PMC4314030

[B49] Thomas R. L. Roberts D. J. Kubli D. A. Lee Y. Quinsay M. N. Owens J. B. (2013). Loss of MCL-1 leads to impaired autophagy and rapid development of heart failure. 27 1365–1377. 10.1101/gad.215871.113 23788623 PMC3701192

[B50] Velagaleti R. S. Pencina M. J. Murabito J. M. Wang T. J. Parikh N. I. D’Agostino R. B. (2008). Long-term trends in the incidence of heart failure after myocardial infarction. 118 2057–2062. 10.1161/CIRCULATIONAHA.108.784215 18955667 PMC2729712

[B51] Wu X. He L. Chen F. He X. Cai Y. Zhang G. (2014). Impaired autophagy contributes to adverse cardiac remodeling in acute myocardial infarction. 9:e112891. 10.1371/journal.pone.0112891 25409294 PMC4237367

[B52] Yamamoto T. Sadoshima J. (2011). Protection of the heart against ischemia/reperfusion by silent information regulator 1. 21 27–32. 10.1016/j.tcm.2012.01.005 22498017 PMC3326382

[B53] Yan L. Vatner D. E. Kim S. J. Ge H. Masurekar M. Massover W. H. (2005). Autophagy in chronically ischemic myocardium. 102 13807–13812. 10.1073/pnas.0506843102 16174725 PMC1224362

[B54] Yang J. Liu X. Bhalla K. Kim C. N. Ibrado A. M. Cai J. (1997). Prevention of apoptosis by Bcl-2: release of cytochrome c from mitochondria blocked. 275 1129–1132. 10.1126/science.275.5303.1129 9027314

[B55] Yuan X. Jing S. Wu L. Chen L. Fang J. (2014). Pharmacological postconditioning with tanshinone IIA attenuates myocardial ischemia-reperfusion injury in rats by activating the phosphatidylinositol 3-kinase pathway. 8 973–977. 10.3892/etm.2014.1820 25120632 PMC4113531

[B56] Zhang S. Tang X. Tian J. Li C. Zhang G. Jiang W. (2011). Cardioprotective effect of sulphonated formononetin on acute myocardial infarction in rats. 108 390–395. 10.1111/j.1742-7843.2011.00676.x 21232020

[B57] Zhang Y. Mi S. L. Hu N. Doser T. A. Sun A. Ge J. (2014). Mitochondrial aldehyde dehydrogenase 2 accentuates aging-induced cardiac remodeling and contractile dysfunction: role of AMPK, Sirt1, and mitochondrial function. 71 208–220. 10.1016/j.freeradbiomed.2014.03.018 24675227 PMC4068748

