# Supplementary material for: Tongguan Capsule Mitigates Post-myocardial Infarction Remodeling by Promoting Autophagy and Inhibiting Apoptosis: Role of Sirt1
Source: Front Physiol. 2018 May 22;9:589. doi: 10.3389/fphys.2018.00589 (PMC5972280; doi:10.3389/fphys.2018.00589)
Supplement: Supplementary file 1 [file Table_1.DOCX]

**Table 1. Echocardiography data performed 6 weeks after MI**

|  | Sham-  vehicle | Sham-  TGC | MI-  vehicle | MI-  TGC | MI-  TGC+3MA | MI-  TGC+EX527 |
| --- | --- | --- | --- | --- | --- | --- |
| Heart rates | 427.41±23.89 | 422.13±27.81 | 492.83±31.66 | 433.88±41.65 | 452.54±51.02 | 462.24±36.24 |
| Systolic Pressure | 117.77±3.23 | 118.09±2.18 | 85.98±4.32 | 101.23±3.11 | 95.65±5.66 | 90.47±3.27 |
| LVEDD | 3.56±0.11 | 3.54±0.18 | 4.98±0.23 | 3.18±0.17 | 3.85±0.18 | 4.12±0.16 |
| LVESD | 1.99± 0.13 | 2.02±0.12 | 4.09±0.19 | 2.16±0.16 | 2.88±0.12 | 3.03±0.21 |
| LVEF | 76.29±2.45 | 75.03±1.98 | 37.01±1.34 | 61.65±1.45 | 50.30±1.23 | 52.24±2.11 |
| LVFS | 44.10±2.19 | 42.94±1.55 | 17.87±0.99 | 32.08±0.69 | 25.19±1.45 | 26.46±1.56 |

LVEDD: LV end-diastolic diameter; LVESD: LV end-systolic diameter; FS: percent fractional shortening; EF: Ejection fraction.
